# Hydrothermal etching fabrication of TiO_2_@graphene hollow structures: mutually independent exposed {001} and {101} facets nanocrystals and its synergistic photocaltalytic effects

**DOI:** 10.1038/srep33839

**Published:** 2016-09-20

**Authors:** Hui Liu, Shuang Liu, Zhiling Zhang, Xiaonan Dong, Tingting Liu

**Affiliations:** 1 School of Materials Science and Engineering, Shaanxi University of Science and Technology, Xi’an 710021, P. R. China

## Abstract

Highly exposed facets TiO_2_ attracts enormous attention due to its excellent separation effect of photogenerated electron-hole pairs and induced high performance of photocatalytic activity. Herein, a novel hydrothermal etching reaction was used to synthesize graphene-wrapped TiO_2_ hollow core-shell structures. Different with the reported co-exposed facets TiO_2_ single crystal nanoparticles, the present TiO_2_ core layer is composed by the mutually independent exposed {001} and {101} facets nanocrystals. Combined with the reduced graphene oxide shell layer, this graphene-wrapped TiO_2_ hollow core-shell structures formed a Z-scheme photocatalytic system, which possess simultaneously the high charge-separation efficiency and strong redox ability. Additionally, the as-prepared samples show a higher absorption property for organic molecules and visible light due to the presence of graphene. All of these unique properties ensure the excellent photocatalytic activity for the graphene-wrapped TiO_2_ hollow structures in the synergistic photo-oxidation of organic molecules and photo-reduced of Cr(VI) process. The TiO_2_ core composed with mutually independent exposed {001} and {101} facets nanocrystals is propose to play an important role in the fabrication of this Z-scheme photocatalytic system. Fabrication of Z-scheme photocatalytic system based on this unique exposed facets TiO_2_ nanocrystals will provides a new insight into the design and fabrication of advanced photocatalytic materials.

Based on the requirement of the increasing energy and environmental-related concerns, photocatalystic technology, which is a light-driven photochemical process over the surface of the photocatalysts and were firstly proposed to producing hydrogen from water, has attracted widely attentions due to its potential applications in the fields of waste water purification, water splitting to hydrogen, biological sensors, solar energy cell, etc^1^,^2^,^3^,^4^. Although the photocatalysis is over-exploited in the past decades, there are still some challenges in this field, such as increasing of the reaction efficiency, inhibiting of the recombination of the photo-generated electrons and holes, even the electrons and holes transport mechanism, etc. All of these points restraining the practical applications significantly.

Recently, enormous interesting is focus on the controlling crystal facets of titania-based photocatalysts due to its fascinating shape-dependent phystalcochemical properties, stable chemical and physical property, excellent photocatalytic performance^5^,^6^,^7^,^8^,^9^,^10^,^11^. It is well know that the average surface energies of anatase TiO_2_ is 0.90 J m^−2^ for {001} > 0.53 J m^−2^ for {100} > 0.44 J m^−2^ for {101} facets, thus, most of synthetic and natural anatase TiO_2_ is inclined to expose {101} facets rather than {001} facets due to higher thermodynamic stability of the former^12^. Because the {001} facets in the equilibrium state has higher surface energy, i.e. higher activity in the photocatalytic process, therefore, in recently a large number of research groups devoted efforts to the synthesis of anatase TiO_2_ exposed {001} facets with high percentages in order to enhance the performances of TiO_2_ nanoparticles in related applications, such as photodegradation of organic pollutants^13^, dye-sensitized solar cells^14^ and water splitting process^15^. Especially, Jiaguo Yu and co-workers proposed a new surface heterojunction concept for well explanation the importance of the optimal ratio of exposed {101} and {001} facets on the enhancement of photocatalytic performance of TiO_2_^16^. However, the most reported TiO_2_-based photocatalysts with exposed facets are on the basis of the single TiO_2_ nanoparticle with co-exposed one high energy facets and other low energy facets. According to the mechanism of the novel Z-scheme photocatalytic system, the major disadvantage of this co-exposed facets system is that the redox ability of photogenerated electrons and holes is weakened^17^. This is mainly because that after charge transfer the photogenerated electrons are concentrated on the conductive band with less positive potential while the holes are centered on the valance band with less negative potential. Although the reported noble metal modified TiO_2_ photocatalysts with co-exposed facets can further promote the separation of the photogenerated electron-hole pairs due to the electron capture center effect of the noble metals. It is still difficult for this TiO_2_ photocatalysts with co-exposed facets by one nanoparticles, which formed the heterojunction-type or surface heterojunction photocatalytic system to a certain extent, to simultaneously possess the high charge-separation efficiency and strong redox ability. Thus, the fabrication of the TiO_2_-based photocatalysts with exposed facets posses simultaneously the high charge-separation efficiency and strong redox ability still remains challenging.

It is well known that the TiO_2_ with exposed facets can produced by adding capping agents^5^,^12^, such as F^−^ and carbonate ion, *et al*. in the synthesis process, and the capping agents play a key role in the expose of the highly energy facets. Herein we report a novel wrapped route and subsequently hydrothermal etching reaction to synthesize graphene-wrapped TiO_2_ hollow core-shell structures. The TiO_2_ core layer of this hollow structure is consists with mutually independent exposed {001} and {101} facets nanocrystals formed by using the hydrothermal etching reaction without adding of capping agents. The photocatalytic activity of the as-prepared graphene-wrapped TiO_2_ hollow core-shell structures were evaluated for synergistic removal effect of organic molecules and Cr(VI) co-exist system in aqueous solution under simulated solar light irradiation, and the possible photocatalytic mechanism of this photocatalyst was also briefly discussed.

## Results and Discussion

**Figure 1:**
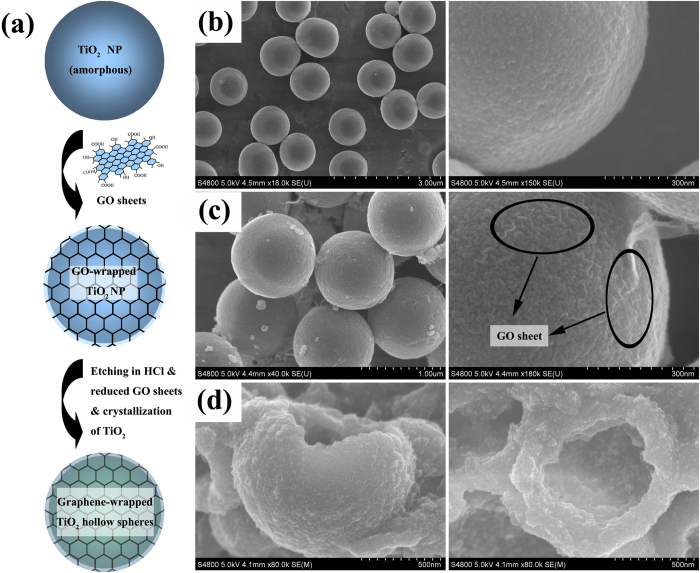
Schematic illustration of synthesis steps for graphene-wrapped anatase TiO_2_ hollow core-shell microspheres (TGHMs) (**a**), and FESEM images of as-prepared pure TiO_2_ microspheres (PT), (**b**), RGO-wrapped amorphous TiO_2_ NPs (**c**) and RGO -wrapped anatase TiO_2_ hollow microspheres (**d**).

The morphology and microstructure of the samples were examined by scanning electron microscopy (SEM) and high-resolution transmission electron microscopy (HRTEM) measurements. Figure 1 displays typical schematic illustration of synthesis steps and corresponding FESEM images of the as-prepared TiO_2_-based composite microspheres. Obviously, the synthesis steps for graphene-wrapped anatase TiO_2_ hollow core-shell microspheres are mainly as follows: firstly, the surface of amorphous TiO_2_ microspheres was directly wrapped with graphene oxide (GO) nanosheets via a chemical bonding reaction, which involved with dehydration condensation between TiO_2_ and GO without any modified agents^18^, followed by a one-step GO reduction and TiO_2_ crystallization process via hydrothermal etching treatment in a HCl solution (Fig. 1a). During the hydrothermal etching treatment process, the amorphous TiO_2_ would transform to anatase phase and meanwhile the GO would be reduced to graphene phase. It can be seen form Fig. 1b, before the warped process, the surface of the pure TiO_2_ microspheres is smooth, and the whole microspheres with the average diameter about 650–700 nm, are composed of large amounts of TiO_2_ nanoparticles. Different from that of the pure TiO_2_ microspheres, we found that the surface of the sample TiO_2_@GO core/shell microspheres present a wrinkle-like morphology (Fig. 1c) after GO wrapped process, which confirms that graphene oxide sheets have combined with the TiO_2_ microspheres. This close interconnection between TiO_2_ and graphene shell is believed to be greatly helpful: i Promotion the transfer and separation of photo-generated electrons from TiO_2_ cores to the graphene shells due to the graphene can be seen as a electron media materials; ii Inhibition the aggregation of the TiO_2_ colloidal microspheres of this core/shell structure in the aqueous solution; iii Avoiding the phase separation during cyclic utilization. While, after the hydrothermal etching treatment of this TiO_2_@GO core/shell microspheres in a HCl solution (Fig. 1d), the graphene-wrapped TiO_2_ hollow core-shell microspheres (TGHMs) can be obtained. It is clearly that the morphology of the graphene oxide wrapped TiO_2_ core-shell microspheres was well maintained, and the dense precursors turned into loose hollow structure. The thickness of this hollow TGHMs layer was determined to be ~150 nm, which may be composed of large amount of TiO_2_ in the hybrid system (Fig. 1d). Furthermore, the corresponding TGA (Figure S1) measurement was carried out to evaluate the weight ratios of the as-prepared TGHMs sample. Because the graphene will be removed when the temperature is higher than 500 °C, thus the loss of quality is mainly belong to the remove of graphene, eliminate the mass loss about 3% within the temperature 100 °C, which can be attributed to the remove of the absorbed water of the sample, the mass percent concentration of graphene in the TGHMs sample is about 17%. It is worth noting that the existence of Cl^−^ and graphene shell play a key role in the formation of this hollow structure. Similar with that of the reported literature^19^, the hollow transformation in this study can be elucidated by the synergetic effect of etching, graphene oxide coating, low hydrothermal reaction temperature, and the unique microstructures of amorphous TiO_2_ precursors composed of amount of nanoparticles. With the confinement of graphene shell and water penetration, the amorphous TiO_2_ precursors are selectively etched and hollowed by chloride ion without destroying their spherical morphology. Meanwhile TiO_2_ hydrates are gradually crystallized again and on the surface of the graphene oxide shell and forming the as-prepared hollow core-shell structure composed of TiO_2_ and graphene oxides. Meanwhile, in order to confirm the importance of the graphene oxide shell in maintaining of the spherical structures, the as-prepared amorphous TiO_2_ precursors were directly hydrothermal treated in HCl solution without the graphene oxides wrapping process. In the absence of graphene oxides coating, hydrothermal treatment of TiO_2_ precursors leads to the formation of mixture of free single crystal TiO_2_ nanoparticles with the diameter about 10–15 nm, as shown in Figure S2.

**Figure 2:**
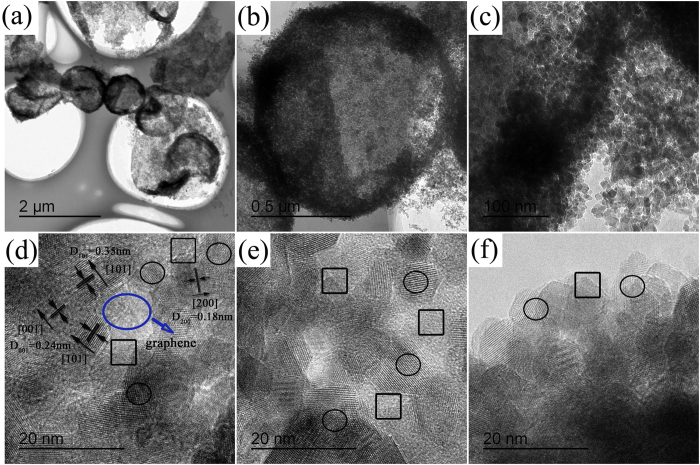
HRTEM images of as-prepared TGHMs. (**a–c**) low- and (**d–f**) high magnification images of TGHMs. The selected area in (**d–f**) give the existence of graphene species (marked with blue) and corresponding (101) facets (selected area as black ellipse) (001) facets (selected area as black box).

The micro/nanostructures of the TGHMs were further investigated by HRTEM measurements, as manifested in Fig. 2. Obviously, it can be seen from Fig. 2a,b, the as-prepared TGHMs revealed a spherical morphology and loose structure. The average diameter of translucent hybrid structures hollow spheres are about 650–700 nm, which is consistent with the SEM measurements (Fig. 1d). Nonetheless, a large number of TiO_2_ nanocrystals with the diameter of 10–20 nm can be seen to distribute rather uniformly on the inner surface of graphene framework (Fig. 2c). The as-prepared TGHMs also presented a clear lattice and the crystal planes {101} and {001} were assigned to anatase TiO_2_ (Fig. 2d)^20^. The HRTEM viewed from the {001} crystallographic direction clearly exhibits the atomic planes of {001} crystal facets with a lattice space of 0.24 nm, supporting that the exposed facets are {001} facets of anatase TiO_2_. The selected areas (i.e., black ellipse represents as {101}, black box represents as {001}) in d (e and f) show different crystal lattices and the {001} facets are always surrounded by {101} facets, which may be similar to that of the surface heterojunction form by the singlecrystal TiO_2_ nanoparticles and favorable to the charge-separation. Meanwhile, the HRTEM image of the product (Figure S2 d), which belong to the directly hydrothermal treated results of the as-prepared amorphous TiO_2_ precursors in HCl solution without the graphene oxides wrapping process, show also the clearly co-existence of {101} and {001} facets, and the corresponding facet space is 0.35 nm and 0.24 nm, respectively. This further approved that the existence of chloride ion may be benefit to the co-expose of {101} and {001} facets of TiO_2_. As we all know, {001} facets with a higher energy (0.90 J/m^2^) than that of {101} facets with the energy (0.44 J/m^2^). Although {001} facets with a higher energy (0.90 J/m^2^) are more interesting and important for higher reactivity, they are usually dominated rapidly by the thermodynamically stable {101} facets (more than 94 percent, according to the Wulff construction) during a crystal growth process^21^,^22^,^23^,^24^,^25^,^26^. Therefore, the long co-existence and gradual exposure of reactive higher energy {001} facets and thermodynamically stable {101} facets may generate numbers of thermal intermediate interfaces, which would be beneficial for reducing the hybrid system Gibbs energy and favorable to enhancing of synergistic photosensitized removal of Cr(VI) and rhodamine B in aqueous solution.

**Figure 3:**
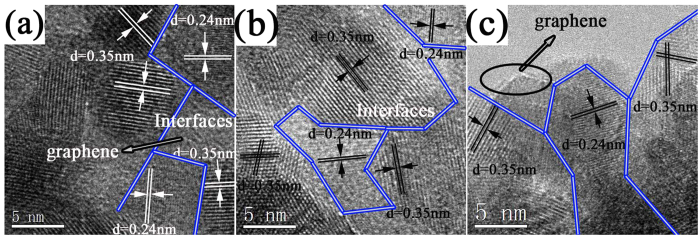
HRTEM images of corresponding facet space of as-prepared TGHMs. The facet space of (101) corresponds to 0.35 nm and the facet space of (001) corresponds to 0.24 nm, respectively.

Figure 3 exhibits the lattice images of the nanoparticles attached on the corresponding selected area mentioned above, including black box and black ellipse (Inset in Fig. 2e,f). Due to the highly crystallinity, the clear crystal lattices and interfaces were all observed in Fig. 3. The lattices images in Fig. 3a,b, which is corresponding to selected area in Fig. 2e, show that the crystal lattice space of 0.35 nm and 0.24 nm ascribed to {101} facet and {001} facet of anatase TiO_2_. Furthermore, the reactive {001} facets are always surrounded by thermodynamically stable {101} facets, and thus generating large numbers of interfaces between each other. In general, two phase interfaces get reactive due to its containing large numbers of reactive species [i.e. various defects]. Therefore, the interfaces between high energy {001} and {101} facets would become reaction fields for synergistic photosensitized removal of Cr(VI) and rhodamine B dye under stimulated solar light irradiation. Besides, the lattice images in Fig. 3c, which is corresponding to selected area in Fig. 2f, also shows that the crystal lattice space of 0.35 nm and 0.24 nm ascribed to {101} facet and {001} facets. In addition, the edge of the graphene can also be observed and indicated in Fig. 3c. This suggested that the exposed {101} and {101} facets of TiO_2_ was attached to the surface of graphene, and thus probably forming some interfaces between TiO_2_ and graphene. Besides, these reactive interfaces are always located in border of composites system, which is also close to graphene species. To some extents, graphene species would play a transport channel role in the photocatalytic reaction because the majority graphene species locates in the gap of {101} and {001} facets. Thus, different with that of the noble metal nanoparticles, which acted as an electrons traps in most of the noble metal modified facets exposed TiO_2_ system, the graphene species in this system not only assumed the duty of electron traps, but transmit the electrons between two mutually independent exposed {101} and {001} facets nanocrystals. Additional, due to its excellent electronic conductivity, mechanical properties and high specific surface area, graphene species could become a bridge to import the whole dye or Cr(VI) molecules before photocatalytic reactions while export the molecular fragments or intermediate product after reactions, and thus reducing the hybrid system Gibbs energy to keep the chemical equilibrium of the whole mixture system better and enhancing the approach of synergistic photosensitized removal of Cr(VI) and rhodamine B dye under stimulated solar light irradiation.

**Figure 4:**
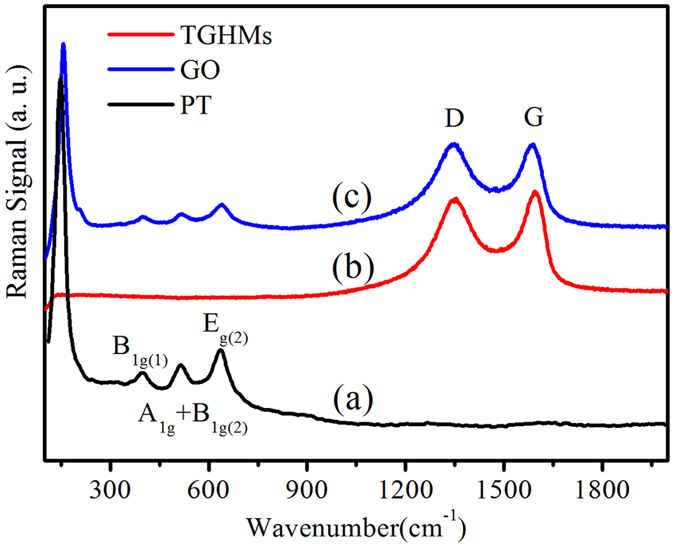
Raman spectra of (**a**) anatase TiO_2_, (**b**) GO, and (**c**) graphene wrapped TiO_2_ hollow core/shell microspheres. In contrast to GO and TiO_2_, the core/shell microspheres contained anatase TiO_2_ and graphene.

The Raman spectrum for TGHMs core/shell structural microspheres (Curve c in Fig. 4) shows several characterized bands at 148, 399, 518, and 639 cm^−1^, which can be attributed to the E_g(1)_, B_1g(1)_, A_1g_ + B_1g(2)_, and E_g(2)_ modes of anatase phase of TiO_2_, respectively^27^. It is worth noting that the intensity of these entire inherent bands weakened seriously, which also confirm the existence of graphene oxide. On the other hand, obviously, two typical bands at around 1343 cm^−1^ (for D band) and 1586 cm^−1^ (for G band) for the graphitized structures were observed. The D band (1343 cm^−1^) is corresponding to disorder carbon while the G band (1586 cm^−1^) is attributed to sp^2^ hybridized carbon^28^,^29^,^30^. This confirmed that the graphene component are maintained during the hydrothermal etching process. In additional, compared with that of the pure graphene oxides (Curve b in Fig. 4), the *I*_*D*_*/I*_*G*_ ratio is obviously increased in the Raman spectrum of the as-synthesized TGHMs sample, which was caused by the reduction of graphene oxide. After the hydrothermal treatment, the oxygen functional groups in the GO were removed, and the conjugated G network (Sp^2^ carbon) will be re-established, and the size of the re-established G network is smaller than the original one, which would lead to an increase in the *I*_*D*_*/I*_*G*_ ratio. Thus the experimental results in this study suggesting a successful reduction of GO to reduced graphene oxide (RGO).

**Figure 5:**
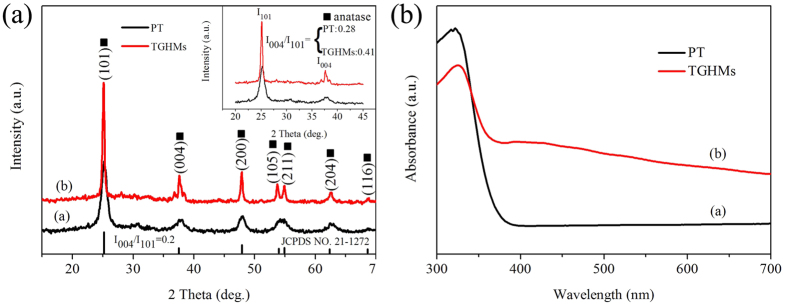
XRD patterns and UV-vis spectra of as-prepared TiO_2_-based composites.

Figure 5a displays the XRD patterns of the as-synthesized samples. Seeing from curve a in Fig. 5a, the X-ray diffraction peaks at 2θ values of 25.2°, 37.9°, 48.1°, 54.1°, 55.0°, 62.7°, 68.8° corresponding to the (101), (004), (200), (105), (211), (204), (116) crystal planes of anatase TiO_2_, respectively, which matched well with reported JCPDS data (Card NO. 21-1272). After HCl hydrothermal etching treatment, the samples revealed much sharper and higher peaks, which might be ascribed to the high crystalline characteristics of TiO_2_ nanocrystals attached on the inner surface of graphene and also implied the formation of TGHMs (curve b in Fig. 5a). Besides, the background of the curves got deeper when the graphene oxide was added to system. To some extent, this phenomenon confirms the combination of carbon species (graphene, etc) with TiO_2_ microspheres^17^,^31^. Because graphene oxide might weak the interaction between TiO_2_ and X-ray light, thus deepening the peaks background of TGHMs composites. In addition, the intensities of the (004) diffraction peaks (0.41 for I_004_/I_101_) are remarkably enhanced as compared with PT (0.28) and the corresponding one of the reference (JCPDS NO. 21-1272) (0.2), suggesting a preferential orientation growth along the {001} facet direction for the TGHMs^32^,^33^. This unique co-existence and gradual exposure of reactive higher energy {001} facets and thermodynamically stable {101} facets may generate numbers of thermal intermediate interfaces, which would be beneficial for reducing the hybrid system Gibbs energy and enhancing the approach of synergistic photosensitized removal of Cr(VI) and rhodamine B dye under stimulated solar light irradiation. Figure 5b reveals the UV-vis absorption spectra of samples (a) PT and (b) TGHMs. The absorption intensity of TGHMs is lower than that of PT in the ultraviolet region, which might be ascribed to the introduction of graphene species. Because graphene species might reduce the quantities of active sites on the surface of semiconductor and weaken the interaction between TiO_2_ and UV light, and thus decreasing the absorption intensity of TiO_2_-based composites in the ultraviolet region. The TGHMs exhibits a larger and stronger absorption band in the range of 400–800 nm, which is attributed to the excellent electron structures of graphene species^34^. Graphene encapsulated the surface of TGHMs have affected the dielectric constant of the surrounding matrix, thus enhancing the visible light absorption of TGHMs^35^,^36^. According to a previous study^37^, the band gaps (Eg) of PT (a) and TGHMs (b) were determined based on the theory of optical absorption for direct band gap semiconductors:


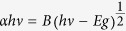


where *hν* are photon energy, and B is a constant related to the material. The value of α can be calculated from the diffuse reflectance data using the Kubelka-Munk function. The band gap of TGHMs (curve b) is smaller than that of PT (curve a in Fig. 5b), which exactly supports our previous assumption (Supporting Information, Figure S3). We further measured the photocurrents under stimulated solar light of the as-prepared TiO_2_-based composites [i.e. PT and TGHMs] deposited on FTO electrodes. A fast and uniform photocurrent response was observed for each switch-on/off event in both TiO_2_-based photocatalysts-deposited electrodes (Figure S4). Under stimulated solar light irradiation, photocurrents of TGHMs composite electrodes were approximately about 6 times higher than that of the PT electrode, which indicates that the separation efficiency of photogenerated electrons and holes was enhanced through the electronic interaction between graphene and TiO_2_. Under a stimulated solar light irradiation, as expected from the results in Figure S4, a lower photocurrent was recorded for PT.

**Figure 6:**
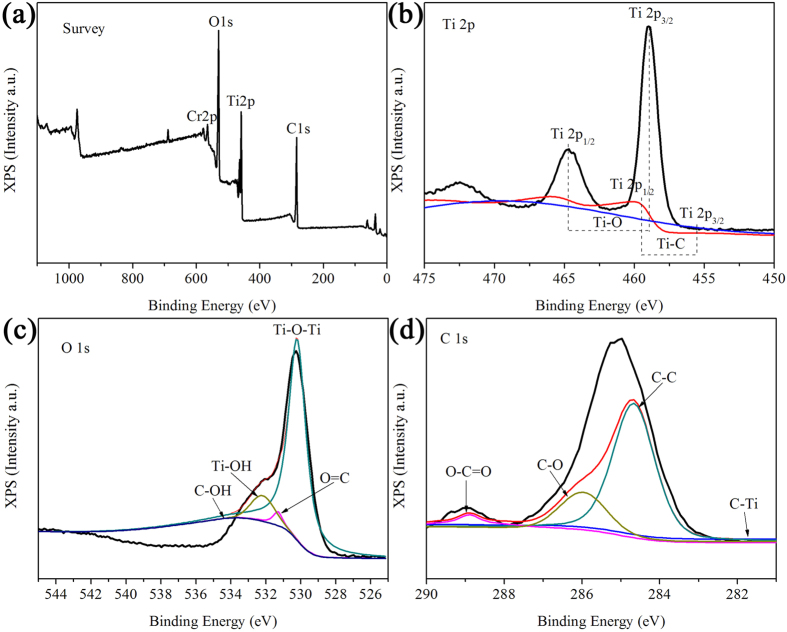
XPS spectra of (**a**) survey, (**b**) Ti 2p, (**c**) O 1 s, and (**d**) C 1 s region for the as-prepared TGHMs (graphene wrapped TiO_2_ hollow core/shell microspheres).

To observe the interaction between graphene and TiO_2_ in TGHMs, FTIR measurement was firstly carried out to the performance of the as-prepared TGHMs samples (Figure S5). It can be seen from Figure S5, compared with that of the pure TiO_2_ microspheres, the FTIR spectrum (Curve b in Figure S5) of the as-prepared TGHMs sample shown a broad absorption peak within lower wavenumber range, this broad absorption peak confirmed that the new chemical bond formed duo to the introduce of the rGO. However, because the probably new formed chemical bond, such as Ti-C or Ti-O-C band, *et al*. will also be located at lower wavenumber range, the already existed broad absorption peak effected the identification of the newly formed chemical bond, thus we further carried the XPS measurement to confirm the interaction between graphene and TiO_2_ in TGHMs. Figure 6 shown the XPS spectra of survey, Ti 2p, O 1 s, and C 1 s region of the as-prepared TGHMs, respectively. The binding energies in all of the obtained XPS spectra were calibrated using C 1 s at 284.6 eV. The survey XPS spectrum of the TGHMs sample (Fig. 6a) exhibits the presence of Ti, O, C and Cr components. Seeing from Fig. 6b, two other weak peaks centered at 455.3 eV and 459.5 eV (involved with the Ti 2p_3/2_ and 2p_1/2_) except for the two characteristic peaks of TiO_2_ at 458.9 eV (Ti 2p_3/2_) and 464.9 eV (Ti 2p_1/2_) were observed and probably ascribed to the Ti-C bond between TiO_2_ and graphene in TGHMs composite. This reveals that the Ti-C chemical bond exists in TGHMs. Figure 6c shows the high resolution spectra O 1 s of TGHMs. With respect to the XPS spectra of O 1 s in Fig. 6c, four peaks at 530.4, 531.2, 532.2 and 533.7 eV have been found, which are corresponding to Ti-O-Ti (lattice O), C=O (and COO), Ti-OH, and C-OH (C-O-C) species, respectively^38^,^39^. Figure 6d exhibits the C 1 s spectra for the as-prepared TGHMs. Two obvious peaks at about 284.6 eV and 286.2 eV corresponding to sp^2^ hybridized carbon in graphene and oxygen bound species C-O bond^40^,^41^. Besides, an obvious peak at about 288.6 eV is observed, corresponding to oxygen bound species O-C=O. In addition, a shoulder-peak located at about 281.1 eV was observed, which was corresponding to the formation of chemical bond between titanium atom in the lattice of TiO_2_ lattice and carbon atom^42^. In order to determine the Cr adsorbed on TGHMs after photoreaction, Figure S6 show the spectrum of XPS of resultant, which proved the existence of Cr(III) species after photocatalytic process^44^, this Cr(III) species is corresponded to the Cr(III) in Cr(OH)_3_^45^,^46^, the deposition of Cr(OH)_3_ on the surface of TGHMs can occupy some photocatalytic active sites of TGHMs to some extent, this is further proved by the following reusability experiment of the as-prepared catalyst.

**Figure 7:**
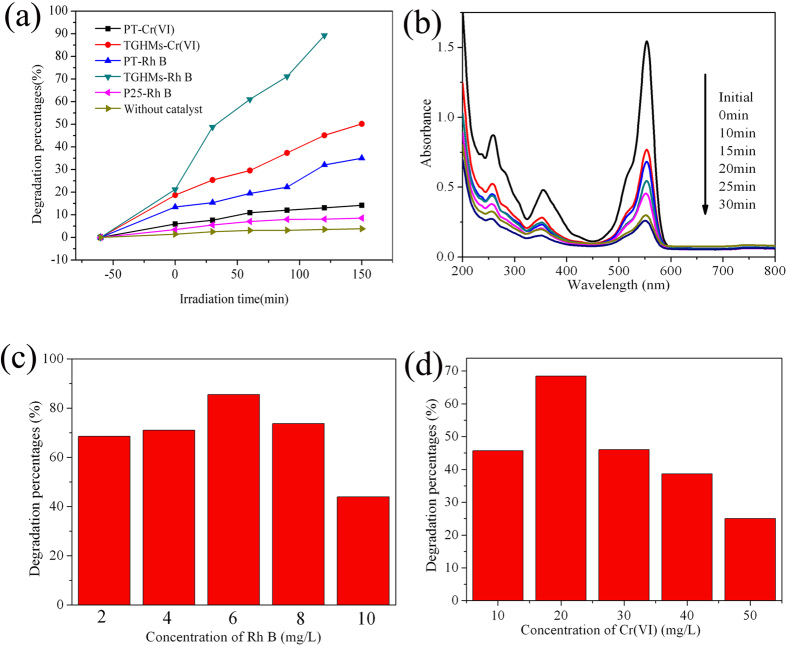
(**a**) photocatalytic conversion of Cr(VI) and Rh B in single system, the initial concentration of Cr(VI) and Rh B is 50 mg/L and 10 mg/L, respectively. (**b**) UV–vis spectra of the synergistic photosensitized removal of Cr(VI) and Rh B dye in aqueous solutions. And the degradation efficiency after 30 min irradiation in mix system for Rh B (**c**) and Cr(VI) (**d**).

To demonstrate the application of fabricated samples [i.e. PT and TGHMs], their photocatalytic activities for synergistic photocatalytic decolorization of Rh B and photoreduction of hexavalent chromium (Cr(VI)) aqueous solution was investigated under stimulated solar light irradiation at ambient temperature. Before the investigation of the photocatalytic reaction in mix system, the photocatalytic activity of the different photocatalysts in single system was performed. Figure 7a shows the photocatalytic conversion of Rh B alone and Cr(VI) alone with P25, PT and TGHMs as the photocatalysts, respectively. It can be seen from Fig. 7a, without catalyst, the dye occurred a self-degradation under stimulated light irradiation, and the degradation rate is about 2–3%, showing that the dye have a weak self-degradation ability. Compared with that of the P25 and PT, the as-synthesized TGHMs shown the higher both reduction ability for Cr(VI) and degradation ability for Rh B in single system. After light irradiation for about 150 min, the conversion rate of TGHMs for Cr(VI) and Rh B achieve to near 50% and 100%, respectively. This can be attributed that the as-synthesized TGHMs composed with mutually independent {101} and {101} facets exposed nanocrystals enhanced effectively charge separation and the redox ability of the photocatalyst. In order to further confirm the effect of the co-exposed facets on the enhancing of the photocatalytic activity, the hollow TiO_2_@rGO core-shell structure, in which the TiO_2_ core layer was only composed of {101} facet exposed nanoparticles (Seen as Figure S7), was synthesized according to our previous study^47^. It can be seen from Figure S7 d, compared with that of the as-prepared TGHMs, the synthesized hollow TiO_2_@rGO core-shell structure with the single {101} facet exposed TiO_2_ layer removal nearly 75% of the Rh B after under stimulated solar light irradiation for 150 min. This experimental result further approved that the mutually independent {101} and {101} facets exposed nanocrystals are benefit to the enhancing of the photocatalytic activity of the as-synthesized TGHMs photocatalyst to a certain extent.

Figure 7b shows the changes in the absorption spectra of the reaction solution, which is a mixed solution consisting of 6 mg/L Rh B and 20 mg/L Cr(VI), exposed to stimulated solar light for various time in the presence of TGHMs. As shown in Fig. 7b, the intensity of the characteristic absorption peaks at 553 nm and 258 nm (or 354 nm) belong to Rh B and Cr(VI) both undergo a fairly large decrease with the light irradiation for about 30 min. And the removal rate of the Rh B and Cr(VI) in this mix system achieved to nearly 85% and 70%, respectively. Compared with that of the single system, the removal rate of the Rh B and Cr(VI) is 50% and 25% after light irradiation for about 30 min, respectively, the as-synthesized TGHMs exhibited a synergistic effect for photosensitized removal of Cr(VI) and RhB dye aqueous solutions. And the further experimental results based on the various mix system (Fig. 7c,d), in which the concentration of Rh B changes from 2 mg/L to 10 mg/L accompany with the concentration of Cr(VI) varies 10 mg/L to 50 mg/L, illustrated that the as-synthesized TGHMs photocatalyst show the synergistic photosensitized removal effect for Cr(VI) and Rh B in mix system. And when the mix solution is consist of 6 mg/L Rh B and 20 mg/L Cr(VI), the as-synthesized TGHMs exhibits a highest synergistic removal efficiency. Meanwhile, in the mixed system, the degradation of Rh B and reduction of Cr(VI) were also the process controlled by pseudo-first-order reactions.

The kinetics constants for Cr(VI) reduction and Rh B degradation by TGHMs in single and mixed system shown in Table S1, it can be seen that when the concentration of Rh B and Cr(VI) is 6 mg/L and 20 mg/L, respectively, the K_app_ value of Rh B and Cr(VI) achieved to the highest value, i.e. 4.47 × 10^−2^ min^−1^ for Rh B and 2.35 × 10^−2^ min^−1^ for Cr(VI), respectively. This was possible due to an increase intermediates adsorbed onto the TGHMs photocatalyst with the increase of Rh B and Cr(VI) concentration, and the active sites would be occupied, as the result, the rate decreased^43^, As a word, the as-synthesized TGHMs photocatalyst in this study present a higher synergistic photosensitized removal of Cr(VI) and Rh B dye in aqueous solutions, this excellent property can first attributed to the unique photocatalytic system build up by the mutually independent exposed {001} and {101} facets nanocrystals and RGO, this structure is favorable to enhancing of charge-separation efficiency and redox ability of the as-synthesized TGHMs photocatalyst. Furthermore, when the photocatalyst was used to a mix system, the degradation of Rh B and reduction of Cr(VI) both effectively consume the separated holes and electrons, respectively, and as the result, the separation efficiency of the electron-hole pairs can be further enhanced. Thus, the photocatalyst shown a higher synergistic photosensitized removal of Cr(VI) and Rh B dye in aqueous solutions.

The stability and recyclability property of the catalyst is crucial for its practical application. In order to evaluate the photostability of TGHMs photocatalyst, we carried out experiments five times to test the recycle use of the photocatalyst by adding fresh Rh B and Cr(VI) solution under stimulated solar light irradiation (with same initial concentration). The experimental results are shown in Figure S8. It can be seen form Figure S8, although there is an indistinct decrease for both photo-oxidation of organic molecules and reduction of Cr(VI) in the reuse photocatalytic process of the TGHMs, it is no obvious performance weakening for the as-prepared TGHMs after five repeated cycles, these results reveal the good stability and reusability of our as-prepared TGHMs photocatalyst. On the other hand, the microstructure of the as-synthesized TGHMs photocatalyst was well maintained after the photocatalytic reaction process (Seen SEM image shown in Figure S9), and this further ensured the good stability and reusability of the photocatalyst.

**Figure 8:**
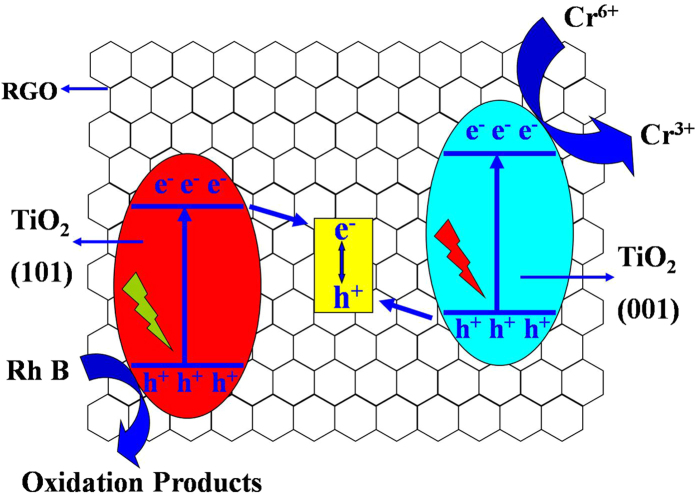
Mechanism of synergistic photosensitized removal of Cr(VI) and Rh B in a Z-scheme photocatalytic system consisting of rGO and mutually independent exposed {001} and {101} facets nanocrystals under stimulated solar light irradiation.

Based on the above experimental, a possible mechanism for the activity of as-synthesized TGHMs based on a Z-scheme photocatalytic redox system was proposed (Fig. 8). In the point of view reported by Rose Amal and co-workers^48^, the RGO in this work can be seen as an electron conductor, which does not contribute to the generation of electrons and holes by absorption of light, it just as a solid-state electron mediator and serves as a pathway for photogenerated electrons. Under light irradiation, electrons are photoexcited from valence band of {001} facets exposed TiO_2_ nanocrystals or {101} facets exposed TiO_2_ nanocrystals to their individual conduction band. Then the RGO provides pathways for the photogenerated electrons from conduction band of {101} facets exposed TiO_2_ nanocrystals (which has a less positive potential) to the valence band of {001} facets exposed TiO_2_ nanocrystals (which has a less negative potential)^17^,^49^, and accomplish a combination process of photogeneratred electrons and holes. Simultaneously, the electrons retained in the conduction band of {001} facets exposed TiO_2_ nanocrystals reduce Cr(VI) to Cr(III), while the holes exist in valence band of {101} facets exposed TiO_2_ nanocrystals oxidize organic molecules to CO_2_ and H_2_O, accomplishing a complete synergistic photosensitized removal of Cr(VI) and Rh B cycle. It is worth mentioning that, unlike the surface heterojuction system form by the co-exposed facets singlecrystal TiO_2_ nanoparticles, in which the electrons transfers to the conduction band with the less positive potential and holes transfers to the valence band with less negative potential duo to existence of the internal electron field formed by the heterojuction structure, it is electrodynamically possible to flow electrons from conduction band of {101} facets exposed TiO_2_ nanocrystals to the valence band of {001} facets exposed TiO_2_ nanocrystals through the RGO pathway due to the existence of the mutually independent facets exposed TiO_2_ nanocrytals in the current system. It is precisely because of the existence of this process, the as-synthesized TGHMs photocatalyst possess simultaneously the high charge-separation efficiency and strong redox ability and guarantee the high performance for synergistic removal of Cr(VI) and Rh B in aqueous solution. Furthermore, it is reported that it is beneficial to the photocatalytic system if the two photosensitive systems produce the same number of photogenerated charge carriers^17^, and this can be achieved by optimize the molar ratio of two different photosensitive systems. In the current photocatalytic system, the two photosensitive systems are all facets exposed TiO_2_ nanocrystals with similar physicochemical properties, and this two photosensitive systems has nearly the same amounts (the {001} facets and {101} facets exposed nanocrystals are always surrounded by the others, seen from Figs 2 and 3, except for the little amount of {200} facets exposed nanocrystals shown in Fig. 2d), this further avoid the recombination of photogenerated charge carriers and enhance the photocatalytic quantum efficiency. As a word, based on the above mentioned reasons, the photocatalytic system in this study bring out an high performance for synergistic photosensitized removal of Cr(VI) and Rh B in aqueous solution.

## Conclusions

The graphene-wrapped TiO_2_ hollow core-shell structure TGHMs photocatalyst was prepared by using a novel direct wrapped route and subsequently hydrothermal etching reaction route. The as-prepared sample possesses simultaneously the high charge-separation efficiency and strong redox ability due to the formation of a Z-scheme photocatalytic system. Additional with the high absorption for organic molecules and solar light, the as-synthesized TGHMs photocatalyst exhibited excellent synergistic photosensitized removal of Rh B and Cr(VI) in aqueous solution. The TiO_2_ core layer composed with mutually independent exposed {001} and {101} facets nanocrystals is propose to play an important role in the fabrication of this Z-scheme photocatalytic system. Fabrication of Z-scheme photocatalytic system based on the mutually independent exposed facets TiO_2_ will provides a new insight into the design and fabrication of advanced photocatalytic materials.

## Experimental section

### Preparation of graphene wrapped TiO_2_ hollow core/shell microspheres

Graphene oxide encapsulated TiO_2_ core/shell microspheres were fabricated as our previous references^18^. Typically, appropriate amount of the as-prepared TiO_2_ microspheres and graphene oxide was added into 40 mL distilled water under stirring condition, then the mixed suspension was magnetic stirred for about 20 hours to finish the encapsulate process. After reaction, the suspension was collected by centrifugation and washed with distilled water for three times. The desired amount of above prepared TGMs were dispersed into a mixed solution of ethanol and HCl solution, then autoclaved in a Teflon-lined stainless steel vessel at 180 °C for 24 h. After the hydrothermal etching reaction, the as-prepared products were dried in a vacuum oven at 60 °C overnight to obtain highly crystalline hollow graphene-TiO_2_ core-shell microspheres, i.e. graphene oxide wrapped TiO_2_ hollow core/shell microspheres (which is designated as TGHMs). To make a comparison, the sample of PT (pure TiO_2_) and TGMs (graphene oxide wrapped TiO_2_ core/shell microspheres) were also prepared. The preparation process of the PT and TGMs were same as the above experimental section. Differently, the corresponding products precursors were also directly autoclaved in a Teflon-lined stainless steel vessel at 180 °C for 24 h, which were marked as PT (pure TiO_2_) and TGMs (graphene oxide wrapped TiO_2_ core/shell microspheres), respectively. As for the synthesized of hollow TiO_2_@rGO core-shell structure with the single {101} facet exposed TiO_2_ layer, the detail experimental method is shown in the supporting information.

### Preparation of TiO_2_-based photocatalysts modified photoanodes

First, a FTO electrode (2 cm × 1) was cleaned by sonicated sequentially for 20 min each in acetone, 10% KOH in ethanol, and distilled water. After washing and N_2_ drying, all the photoanodes [i.e., PT and TGHMs] were prepared by depositing a drop of TiO_2_ colloids (10 uL, 5 mg/mL) to completely cover the FTO surface (the thickness of about 6 μm), followed by drying under infrared lamp (60 °C, 2 h).

### Measurements of photocatalytic activity

Photocatalytic activity of all samples was evaluated and compared by the photocatalytic decolorization of Rh B aqueous solution (10 mg/L) and photoreduction of hexavalent chromium (Cr(VI)) aqueous solution (50 mg/L) under stimulated solar light irradiation at ambient temperature. In a typical experiment: 0.02 g of the samples was dispersed in a 20 mL mixed solution of Rh B solution and Cr(VI) solution in a test tube with a diameter about 2.0 cm. The solution was allowed to reach an adsorption-desorption equilibrium among the photocatalyst. A 500 W Xenon lamp was used as a stimulated solar light source to trigger the photocatalytic reaction. After irradiation for an appropriate interval, the reaction solution was filtrated and the corresponding concentration of residual mixed solution of Rh B and Cr(VI) were determined by an UV-visible spectrophotometer (Lambda-950, Perkin Elmer, USA).

### Sample Characterization

The morphology of the as-prepared samples were characterized with a field-emission scanning electron microscope (Hitachi S4800, Japan) operated at 5 KV and a transmission electron microscope (JEM 2010 from JEOL, Japan) operated at 200 KV. The crystalline phase of the products were characterized using X-ray diffraction (XRD-D/max2200pc, Japan) technique with Cu kα radiation of wavelength λ = 0.15418 nm. Raman spectra were recorded at room temperature using a micro-Raman spectrometer (Renishaw InVia) in the back scatting geometry with 514.5 nm Ar^+^ laser as an excitation source. UV-visible absorbance spectra were obtained for the dry-pressed disk samples with a UV-visible spectrophotometer (Lambda-950, PerkinElmer, USA). BaSO_4_ was used as a reflectance standard in a UV-visible diffuse reflectance experiment. X-ray photoelectron spectroscopy (XPS) measurements were performed by using an ultrahigh vacuum VG Scientific Corp MK-II electron spectrometer equipped with a multichannel detector. The spectra were excited using Mg Ka (1253.6 eV) radiation (operated at 200 W) of a twin anode in the constant analyzer energy mode with a pass energy of 50 eV. Fourier transform infrared (FT-IR) spectrometer (JASCO FT/IR-470, Japan) was used for detection of the information of the chemical bonds of the as-prepared samples. Thermogravimetric analyses (TGA) were conducted on a thermogravimetric analyzer, TGA2050 (DTG-60AH SHIMADZU, Japan), with an air flow rate of 30 mL/min at a heating rate of 10 K/min. Photocurrent experiments were performed at room temperature with a CHI 660D workstation (CH Instruments, Chenhua Co., Shanghai, China). A conventional three-electrode cell was used, using an Ag/AgCl electrode (saturated KCl) as the reference electrode, a platinum wire as the counter electrode, and the as-prepared TiO_2_-based photocatalysts modified FTO electrode as a photoanode. A xenon lamp with a 420 nm cut-off filter is used as light source.

## Additional Information

**How to cite this article**: Liu, H. *et al*. Hydrothermal etching fabrication of TiO_2_@graphene hollow structures: mutually independent exposed {001} and {101} facets nanocrystals and its synergistic photocaltalytic effects. *Sci. Rep.* **6**, 33839; doi: 10.1038/srep33839 (2016).

## Supplementary Material

Supplementary Information
