# Supplementary material for: Hydrothermal etching fabrication of TiO2@graphene hollow structures: mutually independent exposed {001} and {101} facets nanocrystals and its synergistic photocaltalytic effects
Source: Sci Rep. 2016 Sep 20;6:33839. doi: 10.1038/srep33839 (PMC5028751; doi:10.1038/srep33839)
Supplement: Supplementary Information [file srep33839-s1.doc]

**Hydrothermal etching fabrication of TiO_2_@graphene hollow structures: mutually independent exposed {001} and {101} facets nanocrystals and its synergistic photocaltalytic effects**

Hui Liu*, Shuang Liu, Zhiling Zhang, Xiaonan Dong, and Tingting Liu

School of Materials Science and Engineering, Shaanxi University of Science and Technology, Xi’an 710021, P. R. China

*Corresponding author. Tel: +86-29-86177018; Fax: +86-29-86177018.

Email address: [liuhui@sust.edu.cn](mailto:liuhui@sust.edu.cn) (H Liu)

**supplementary materials:**

**Fig. S1 (on page 3)** TGA curves of the as-prepared TGHMs sample.

**Fig. S2 (on page 4)** TEM images of as-prepared TiO_2_ nanoparticles with different magnifications.

**Fig. S3 (on page 5)** Plots of (F(R∞)hν) versus(hν) for obtaining the band gaps of (a) PT and (b) TGHMs.

**Fig. S4 (on page 6)** Photocurrent responses of PT and TGHMs under stimulated solar light irradiation.

**Fig. S5 (on page 7)** FTIR spectra of (a) pure TiO_2_ microspheres and (b) as-prepared TGHMs sample.

**Fig. S6 (on page 8)** XPS spectra of Cr 2p after photocatalytic process.

**Fig. S7 (on page 9)** SEM images of the hollow TiO_2_ (a) and TiO_2_@rGO (d) spheres, TEM images TiO_2_@rGO spheres with different magnifications (c-e), and photocatalytic conversion of Rh B (10 mg/L) of hollow TiO_2_ (a) and TiO_2_@rGO (d) spheres (f), respectively.

**Fig. S8 (on page 10)** Photocatalytic performances of TGHMs in the first five reuse cycles.

**Fig. S9 (on page 11)** SEM image of as-prepared TGHMs after photocatalytic performances.

**Table S1** **(on page 12)** Kinetics constants for Cr(Ⅵ) reduction and Rh B degradation by TGHMs in single and mix system.


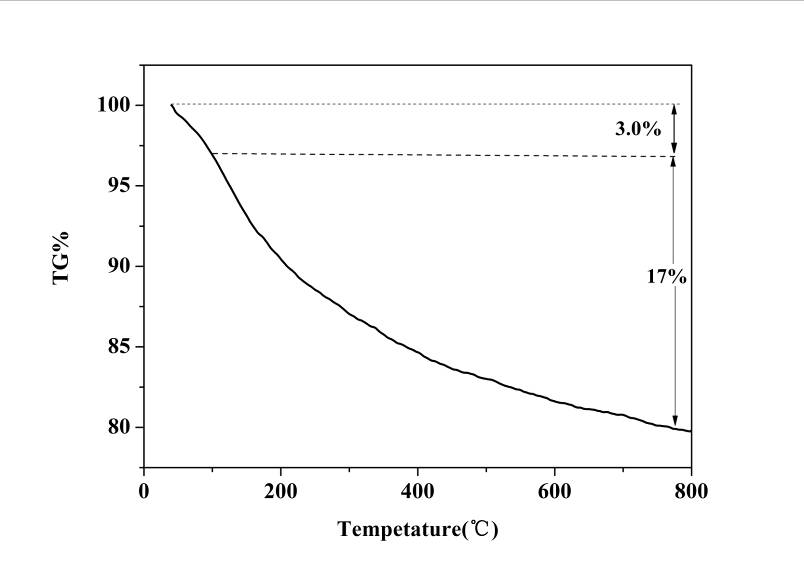


**Figure S1** TGA curves of the as-prepared TGHMs sample.


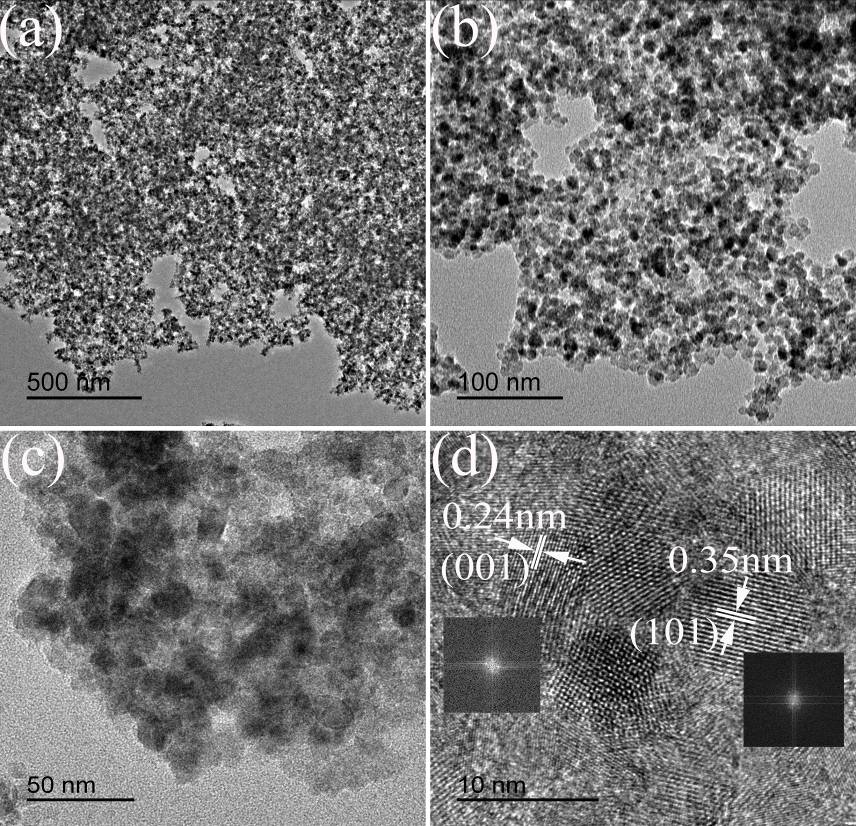


**Figure S2** TEM images of as-prepared TiO_2_ nanoparticles with different magnifications.


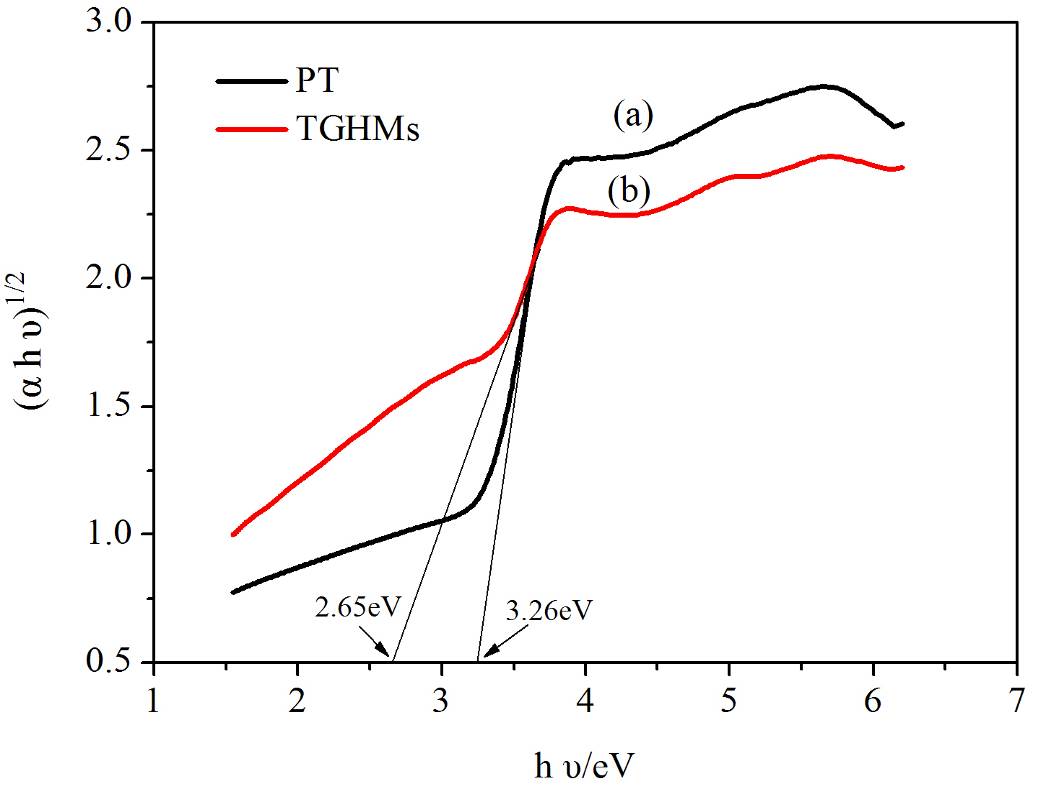


**Figure S3** Plots of (F(R∞)hν) versus(hν) for obtaining the band gaps of

(a) PT and (b) TGHMs.

**
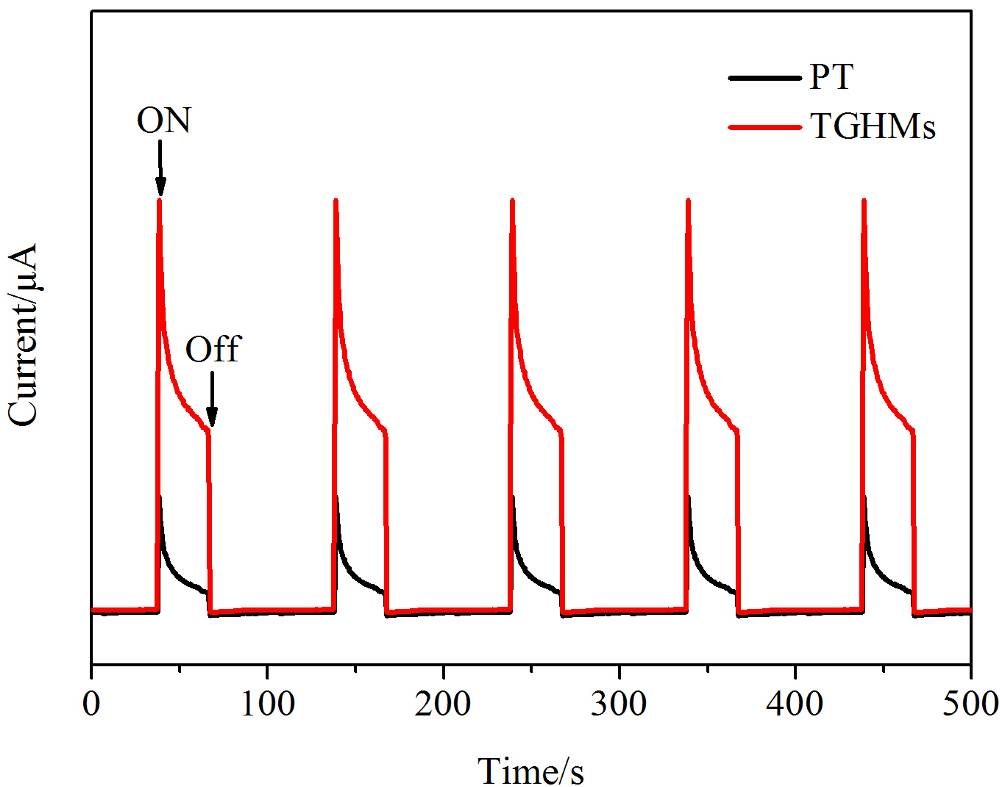
**

**Figure S4** Photocurrent responses of PT and TGHMs under stimulated solar light irradiation.


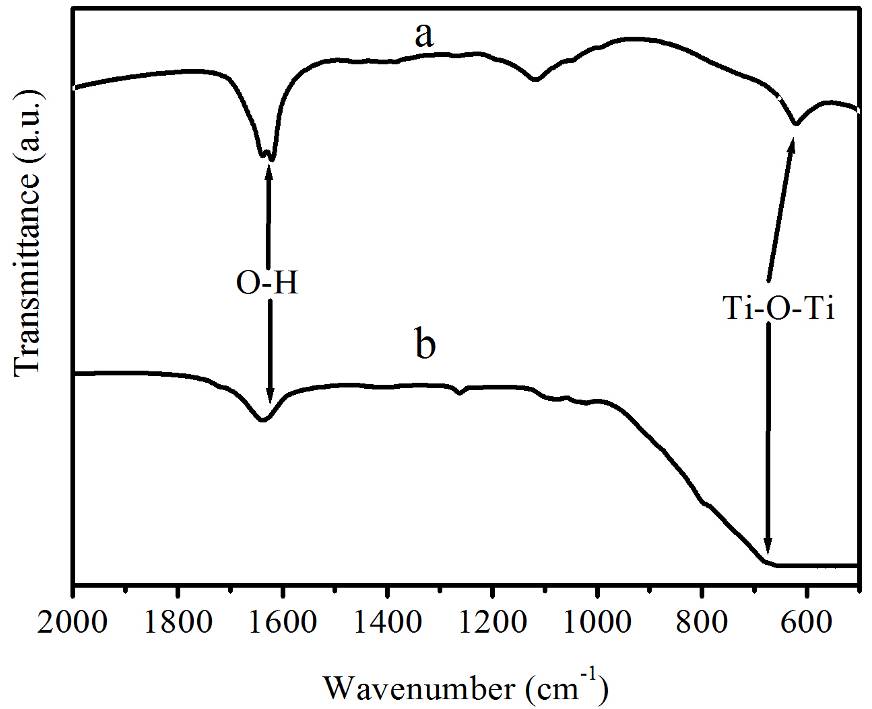


**Figure S5** FTIR spectra of (a) pure TiO_2_ microspheres and (b) as-prepared TGHMs sample.


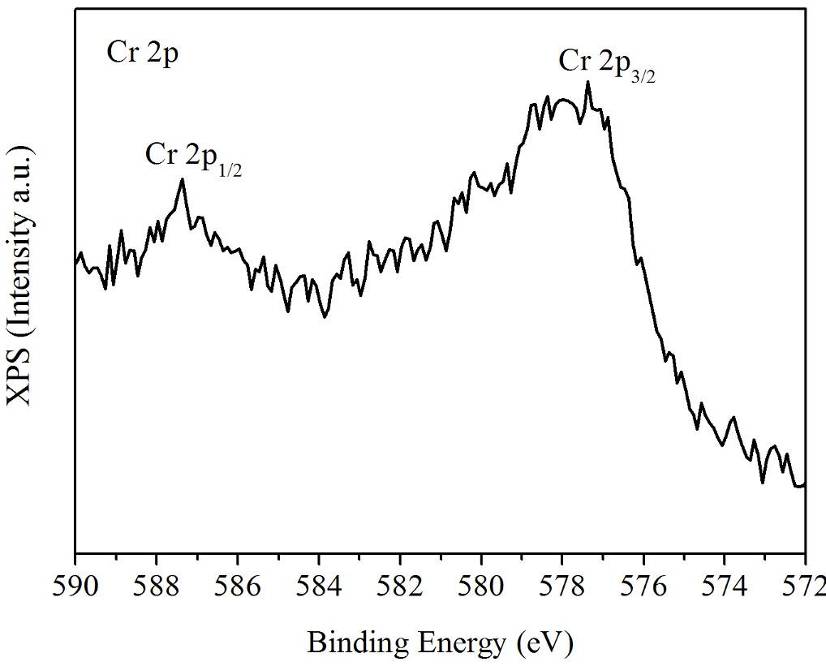


**Figure S6** XPS spectra of Cr 2p after photocatalytic process.


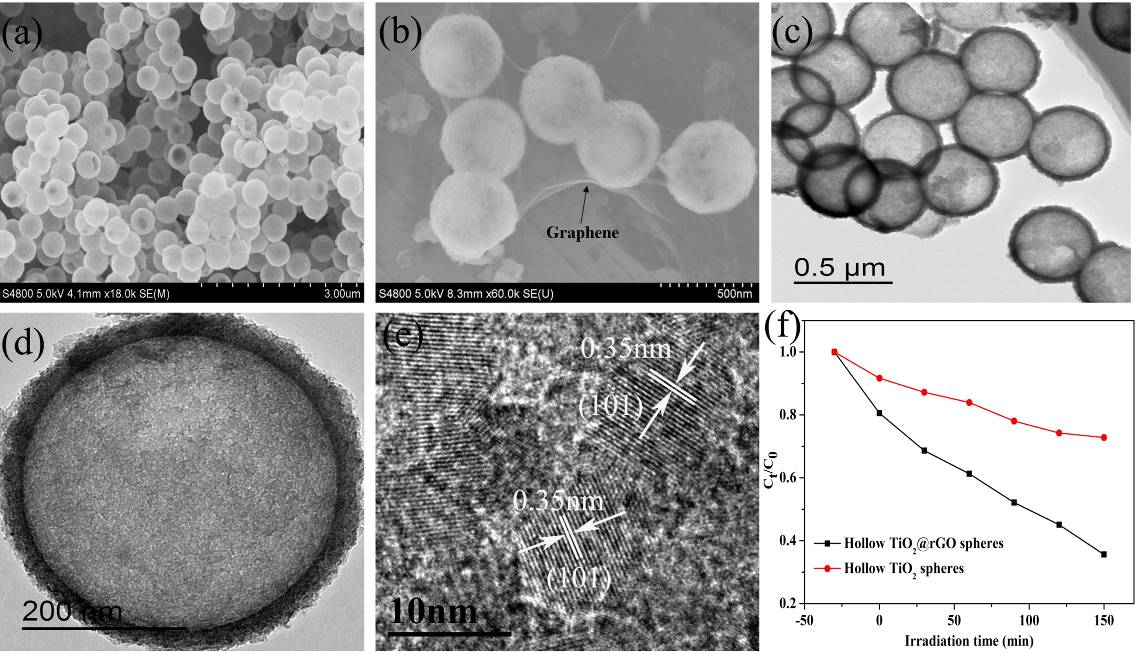


**Figure S7** SEM images of the hollow TiO_2_ (a) and TiO_2_@rGO (d) spheres, TEM images TiO_2_@rGO spheres with different magnifications (c-e), and photocatalytic conversion of Rh B (10 mg/L) of hollow TiO_2_ (a) and TiO_2_@rGO (d) spheres (f), respectively.

The synthesis procedure of the reduced graphene wrapped hollow TiO_2_ core-shell structure is reported in our previous study,^[1]^ the whole process is as follows: firstly, monodispersed SiO_2_ spheres were prepared by using a slightly modified Stöber process. Then, the TiO_2_ nanoparticles were coated on the surface of the SiO_2_ spheres through a hydrolysis process of TBOT. Thirdly, the graphene oxides wrapped composite SiO_2_@TiO_2_@GO spheres were synthesized through a direct dehydration condensation process.^[2]^ Finally, to obtain the reduced graphene wrapped hollow TiO_2_ core-shell structure, the SiO_2_ core was chemically etched with NaOH solution. Different with the reported study, ^[1]^ in the second step, 3 mL of TBOT was added, and the final reduced graphene wrapped hollow TiO_2_ core-shell structure was treated by using a traditional hydrothermal method at 180 ℃ for 24 h in order to obtain a well crystallization product.

[1] Liu, H., Lv, T., Zhu, Z. F.. Template-assisted synthesis of hollow TiO_2_@rGO core–shell structural nanospheres with enhanced photocatalytic activity, J. Mol. Catal. A: Chem. 404, 178–185 (2015).

[2] Liu, H., Dong, X. N., Wang X. C., et al. A green and direct synthesis of graphene oxide encapsulated TiO_2_ core/shell structures with enhanced photoactivity, Chem. Engin. J. 230, 279-285 (2013).


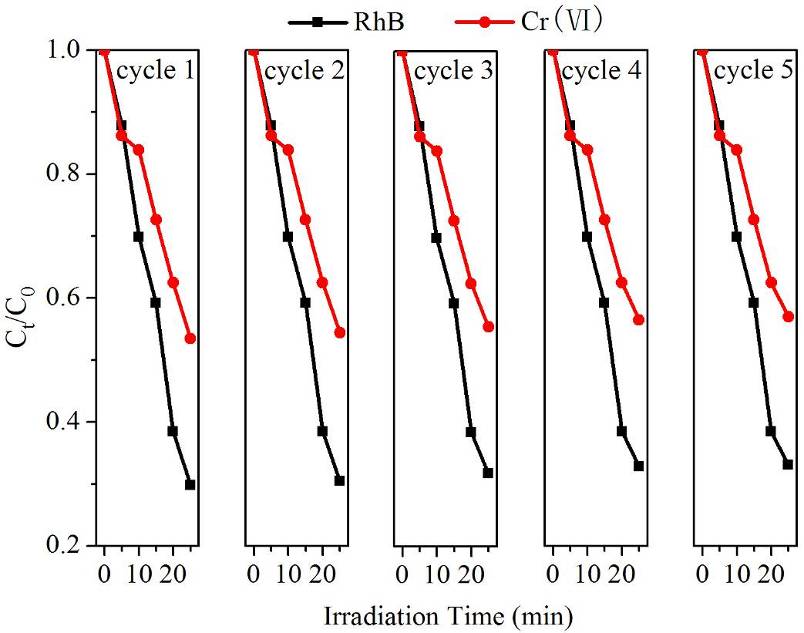


**Figure S8** Photocatalytic performances of TGHMs in the first five reuse cycles.


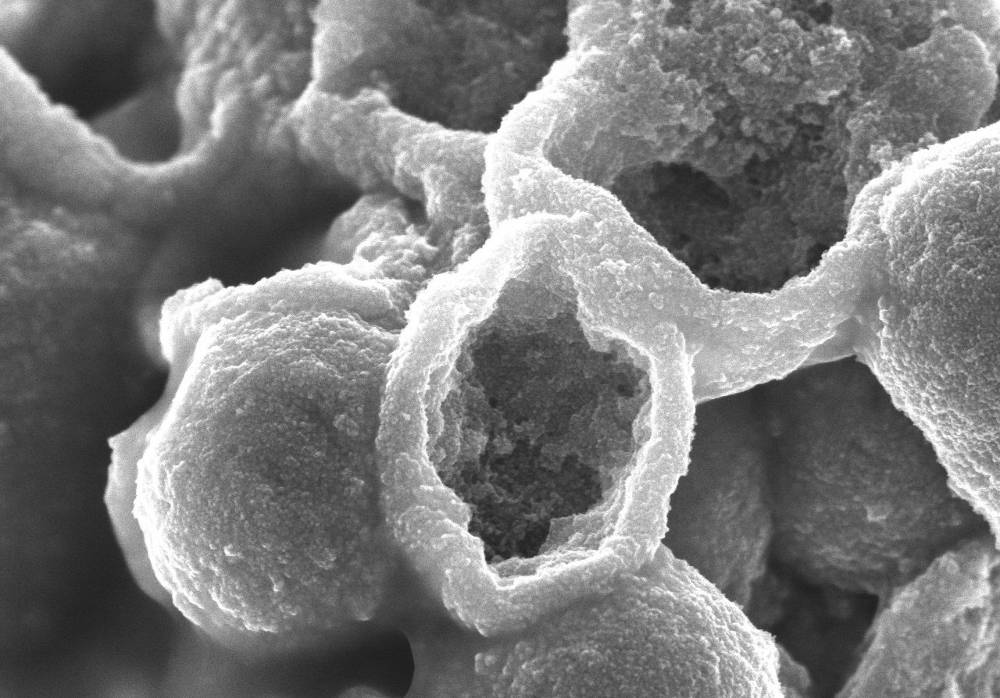


**Figure S9** SEM image of as-prepared TGHMs after photocatalytic performances.

**Table S1** Kinetics constants for Cr(Ⅵ) reduction and Rh B degradation by TGHMs in single and mix system.

| System | k_app_(min^-1^) | | R^2^ | |
| --- | --- | --- | --- | --- |
|  | Rh B | Cr(VI) | Rh B | Cr(VI) |
| 10mg/L Rh B | 1.23×10^-2^ |  | 0.98712 |  |
| 50mg/L Cr(VI) |  | 4.54×10^-3^ |  | 0.92736 |
| 8mg/L Rh B-10 mg/L Cr(VI) | 1.36×10^-2^ | 9.35×10^-3^ | 0.94707 | 0.98386 |
| 6mg/L Rh B-20 mg/L Cr(VI) | 4.47×10^-2^ | 2.35×10^-2^ | 0.96884 | 0.97456 |
| 5mg/L Rh B-25 mg/L Cr(VI) | 1.94×10^-2^ | 6.37×10^-3^ | 0.98743 | 0.98267 |
| 4mg/L Rh B-30 mg/L Cr(VI) | 1.60×10^-2^ | 5.48×10^-3^ | 0.98093 | 0.98101 |
| 2mg/L Rh B-40 mg/L Cr(VI) | 1.40×10^-2^ | 4.61×10^-3^ | 0.80224 | 0.91246 |
